# Anticancer Activities of Hesperidin via Suppression of Up-Regulated Programmed Death-Ligand 1 Expression in Oral Cancer Cells

**DOI:** 10.3390/molecules26175345

**Published:** 2021-09-02

**Authors:** Benjawan Wudtiwai, Anupong Makeudom, Suttichai Krisanaprakornkit, Peraphan Pothacharoen, Prachya Kongtawelert

**Affiliations:** 1 Thailand Excellence Center for Tissue Engineering and Stem Cells, Department of Biochemistry, Faculty of Medicine, Chiang Mai University, Chiang Mai 50200, Thailand; Benjawanwudtiwai@gmail.com (B.W.); peraphan.pothacharoen@gmail.com (P.P.); 2 Center of Excellence in Oral and Maxillofacial Biology, Department of Oral Biology and Diagnostic Sciences, Faculty of Dentistry, Chiang Mai University, Chiang Mai 50200, Thailand; mommam_bom@hotmail.com (A.M.); suttichai.k@cmu.ac.th (S.K.); 3 School of Dentistry, Mae Fah Luang University, Chiang Rai 57100, Thailand

**Keywords:** cell migration, cell proliferation, oral cancer, programmed death-ligand 1, signal transducer and activator of transcription

## Abstract

Up-regulated expression of programmed death-ligand 1 (PD-L1) by interferon-gamma (IFN-γ) has been associated with promotion of cancer cell survival and tumor cell escape from anti-tumor immunity. Therefore, a blockade of PD-L1 expression can potentially be used as a molecular target for cancer therapy. The aim of this study was to investigate whether suppression of IFN-γ induced PD-L1 expression in two oral cancer cell lines, HN6 and HN15, by hesperidin effectively decreased cell proliferation and migration. Further, our objective was to elucidate the involvement of the signal transducer and activator of transcription 1 (STAT1) and STAT3 in the inhibition of induced PD-L1 expression by hesperidin. Our findings indicate that IFN-γ induced expression of PD-L1 protein in HN6 and HN15 via phosphorylation of STAT1 and STAT3 and that hesperidin significantly reduced that induction through suppression of phosphorylated STAT1 and STAT3 in both cell lines. Moreover, hesperidin also significantly decreased the viability, proliferation, migration, and invasion of both cell lines. In conclusion, hesperidin exerted anticancer effects against oral cancer cells through the suppression of PD-L1 expression via inactivation of the STAT1 and STAT3 signaling molecules. The findings of this study support the use of hesperidin as a potential adjunctive treatment for oral cancer.

## 1. Introduction

Oral cancer, ranked the sixth most commonly diagnosed form of cancer, continues to emerge as a major concern in various regions of the world [1]. Oral squamous cell carcinoma (OSCC) is recognized as the most common type of oral cancer [2]. The main risk factors for OSCC are associated with tobacco use and alcohol consumption [3]. However, several other factors may also favor its development and progression such as infections with human papillomavirus and poor oral hygiene [4]. In OSCC, there is a relatively high incidence of invasion into the underlying tissue and/or potential metastasis to distant organs via the lymphatic vasculature, which is known to lead to tumor recurrence and patient morbidity and mortality [5]. Therefore, there is a five-year survival rate associated with OSCC if the cancer is detected at a later stage [6]. Consequently, it would be beneficial to identify a novel adjunctive agent that can be used in combination with standard chemotherapeutic drugs to enhance the treatment efficacy of OSCC.

Interferon-gamma (IFN-γ) is a main cytokine that is produced and secreted by several types of immune cells including T lymphocytes and natural killer cells present in the tumor microenvironment [7]. In melanoma, IFN-γ induces the expression of programmed cell death-ligand 1 (PD-L1) via activation of Janus kinase JAK1 and JAK2, signal transducer and activator of transcription 1 (STAT1), and interferon regulatory factor 1 [8]. Moreover, STAT3 can be induced by IFN-γ as an atypical signal transducer, while a cross-talk relationship between STAT1 and STAT3 has been demonstrated [9]. IFN-γ also induces the expression of PD-L1 in other cancer cell types, including those of oral cancer [10], breast cancer [11], and lung cancer [12], leading to a mechanism known as the immune evasion of cancer cells [13]. A comprehensive understanding of up-regulated PD-L1 expression by IFN-γ, and its associated functions in the aggressiveness of oral cancer cells may, thus, contribute to the development of certain necessary strategies that could maximize the anticancer activities of newly identified agents.

PD-L1 is a transmembrane protein that is expressed in several somatic cells such as antigen-presenting cells. It is a primary ligand for programmed cell death protein-1 (PD-1), a membrane-bound receptor of activated T cells. The binding that occurs between PD-L1 and PD-1 negatively regulates T-cell activation by inducing T cell apoptosis and subsequently acting as a negative immune checkpoint [14,15]. Many cancer cell types exploit this mechanism in order to evade the host immune system for their survival by overexpressing PD-L1 protein on the cell surface. High degrees of PD-L1 expression also benefit cancer cells by enhancing their resistance to chemotherapy and supporting their metastatic ability. Several previous studies [16] have shown that PD-L1 and PD-1 are potential targets for the development of new drugs for cancer treatments. Therefore, any studies that identify the natural compounds that can inhibit PD-L1 signaling in oral cancer cell lines are of significant interest with regard to the search for viable treatment options that could address the growing problem of oral cancer worldwide.

The present study focused on hesperidin, an active compound in orange peel and other citrus species, that is widely used in Chinese herbal medicine [17]. Hesperidin has displayed a potent anticancer effect against various cancer cell lines including prostate cancer, bladder cancer, hepatocellular carcinoma, and breast cancer [18,19,20]. It has been reported that hesperidin exerts its anticancer activity by promoting apoptosis [21], while inhibiting the invasion and metastasis of lung cancer and hepatocellular carcinoma [22]. It is particularly noteworthy to mention that hesperidin has been shown to not be toxic to normal cells [21]. A previous study has suggested that hesperidin may act as an immune checkpoint inhibitor that can suppress expression of the PI3K/AKT pathway in the breast cancer cell line, MDA-MB231 [23]. Consequently, it has been hypothesized that hesperidin may also act as an immune checkpoint inhibitor in oral cancer cells by targeting PD-L1 expression that has been induced by treatment with IFN-γ via suppression of the STAT1/STAT3 signaling pathway, which ultimately can result in the down-regulation of PD-L1 expression and a decrease in the degree of aggressiveness of cancer cells.

## 2. Results

### 2.1. PD-L1 Expression in Oral Cancer Cells

**Figure 1:**
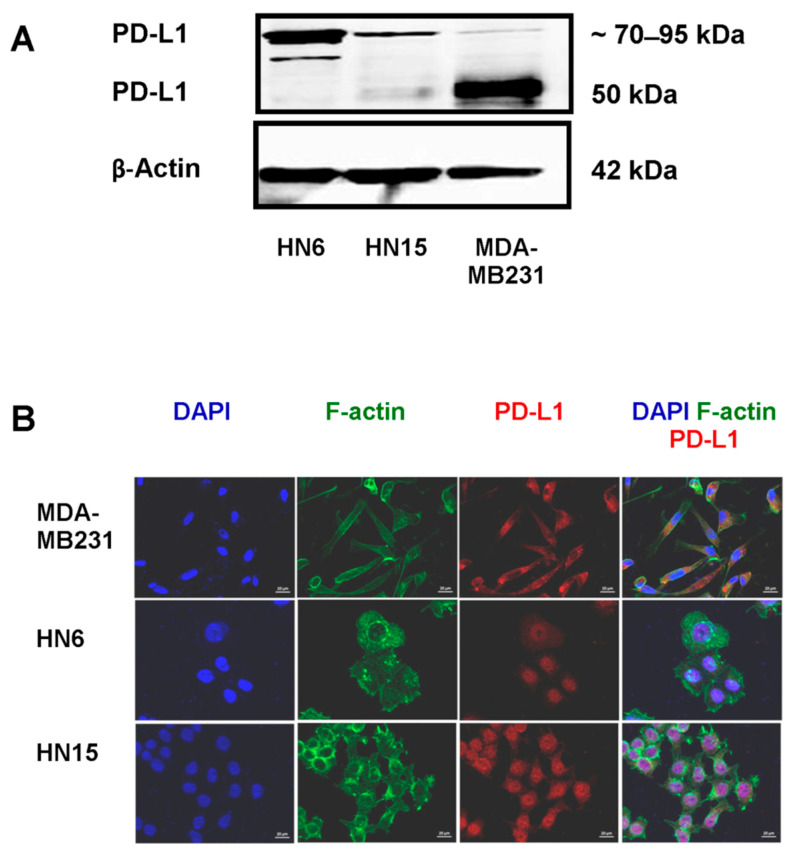
Determination of programmed death-ligand1 (PD-L1) expression in two oral cancer cell lines, HN6 and HN15. (**A**) Immunoreactive bands at around 70–95 kDa in the whole cell lysates of HN6 and HN15 differ from an intense band of PD-L1 at the expected size of 50 kDa in the breast cancer cell line, MDA-MB231. (**B**) Cytoplasmic localization of PD-L1 (red) in the MDA-MB231 cell line was demarcated by staining with anti-F-actin antibody (green), while immunostaining (red) within the nuclei of HN6 and HN15, as indicated by staining with DAPI (blue), was found to be an artifact. Representative findings from three separate experiments are shown. Bars = 200 µm.

By immunoblotting, a faint immunoreactive band of PD-L1 expression at the predicted size (50 kDa) was found in HN15 but not in HN6, whereas an intense band at the same molecular weight was detected in the breast cancer cell line, MDA-MB231, that was used as a positive control [23] (Figure 1A). Note that additional bands or a weak band detected at around 70–95 kDa in HN6 and HN15, or in MDA-MB231, respectively, were consistent with the ubiquitinated PD-L1 proteins that had been previously reported at a molecular weight greater than 64 kDa [24]. Expression of β-actin was equal among the whole cell lysates of these three different cell lines. By immunofluorescence, cytoplasmic localization of PD-L1 protein was not detected in either HN6 or HN15, whereas an intense signal (red) of PD-L1 protein was found in the cytoplasm of the MDA-MB231 cell line (Figure 1B). Note that nuclear staining in HN6 and HN15 (Figure 1B) is considered an artifact as has been previously reported [25].

### 2.2. Up-Regulation of PD-L1 Expression by IFN-γ in Oral Cancer Cells

**Figure 2:**
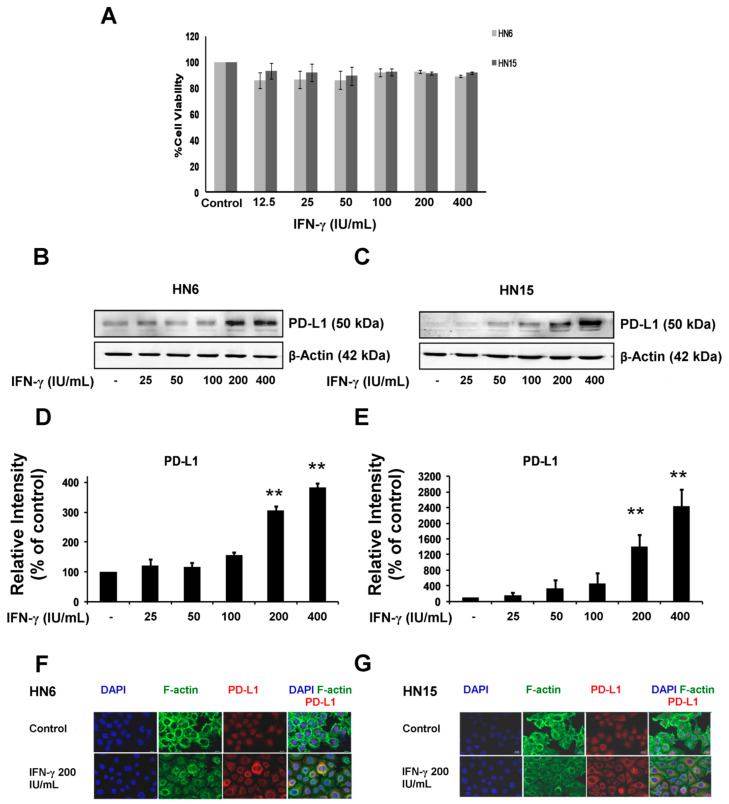
Induction of PD-L1 expression by interferon-gamma (IFN-γ) in oral cancer cells. (**A**) Percentage of cell viability was observed upon treatment with IFN-γ at indicated doses (12.5–400 IU/mL) for 24 h in HN6 (light gray) and HN15 (dark gray) as assessed by the MTT assay. (**B**,**C**) Representative images of up-regulated PD-L1 expression clearly shown by treatment with IFN-γ at 200 or 400 IU/mL for 24 h in HN6 and HN15, respectively. (**D**,**E**) Bar graphs demonstrate the relative intensities of PD-L1 to those of β-actin in IFN-γ-treated HN6 and HN15, respectively. These were compared to those of the untreated cells whose ratio was set to 100%. Data in (**A**,**D**,**E**) are presented as mean ± SD values (error bars) obtained from three separate experiments. ** *p* < 0.01. (**F**,**G**) Representative images from three experiments in HN6 and HN15 that were treated with IFN-γ at 200 IU/mL for 24 h. Note the cytoplasmic localization of PD-L1 (red) upon treatment with IFN-γ, while an artifact of nuclear staining was still found in the control untreated HN6 and HN15 cell lines as indicated by staining with DAPI (blue).

The viability of HN6 and HN15 upon treatment with IFN-γ at indicated concentrations for 24 h was first checked using MTT assay. There was no difference in the mean percentage values of cell viability in HN6 or HN15 upon treatment with doses of IFN-γ up to 400 IU/mL when compared to that of the control untreated cells. This indicated that IFN-γ treatment was not toxic to both cancer cell lines (Figure 2A). Next, the effect of treatment with IFN-γ at different doses (0–400 IU/mL) for 24 h on PD-L1 protein expression in both cell lines was examined. Treatment with IFN-γ induced PD-L1 expression in a dose-dependent manner in both cell lines (Figure 2B,C). Notably, expression of β-actin was equivalent among the different samples. By densitometry, treatment with IFN-γ at 200 or 400 IU/mL significantly enhanced the mean percentage values of PD-L1 expression in both HN6 and HN15 (*p* < 0.01; Figure 2D and Figure 2E, respectively). Moreover, treatment with IFN-γ at 200 IU/mL for 24 h enhanced PD-L1 expression (red) in the cytoplasm of both HN6 and HN15 when compared to nuclear staining in the control untreated HN6 and HN15 cell lines (Figure 2F and Figure 2G, respectively). The cytoplasmic presence of PD-L1 protein by IFN-γ treatment in HN6 and HN15 corresponded with that found in the MDA-MB231 cell line (Figure 1B). Thus, the concentration of IFN-γ at 200 IU/mL was selected for oral cancer cell stimulation in subsequent experiments.

### 2.3. Enhancement of Phosphorylated STAT1 and STAT3 Levels by IFN-γ

**Figure 3:**
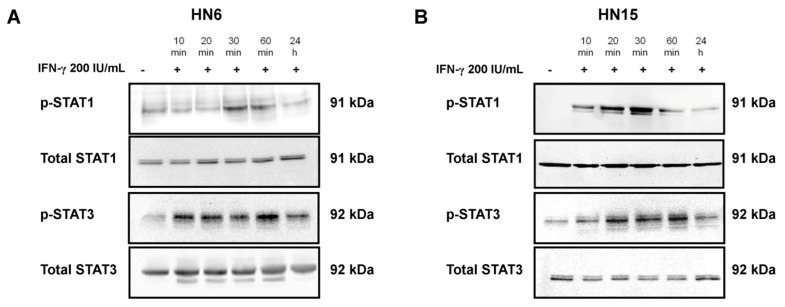
Transient phosphorylation of STAT1 and STAT3 by IFN-γ treatment in two oral cancer cell lines. Representative immunoblots from three separate experiments of phosphorylated-STAT1 (p-STAT1) and STAT3 (p-STAT3) are presented, along with those of total STAT1 and STAT3 in the whole cell lysates of HN6 (**A**) and HN15 (**B**) treated with IFN-γ at 200 IU/mL for indicated times.

To determine whether treatment of HN6 and HN15 with IFN-γ phosphorylated STAT1 and STAT3, two major signaling molecules that mediated the effect of IFN-γ in other cell types [9] were treated with IFN-γ at 200 IU/mL for indicated times. The results revealed that the levels of phosphorylated-STAT1 (p-STAT1) and p-STAT3 were transiently increased in HN6 and HN15 upon treatment with IFN-γ with a salient increase observed at 30 min in both cell lines (Figure 3A and Figure 3B, respectively). Therefore, the inhibitory effect of hesperidin on the phosphorylation of STAT1 and STAT3 was later studied after treatment with IFN-γ for 30 min.

### 2.4. Suppression of the Viability of Oral Cancer Cells by Hesperidin

**Figure 4:**
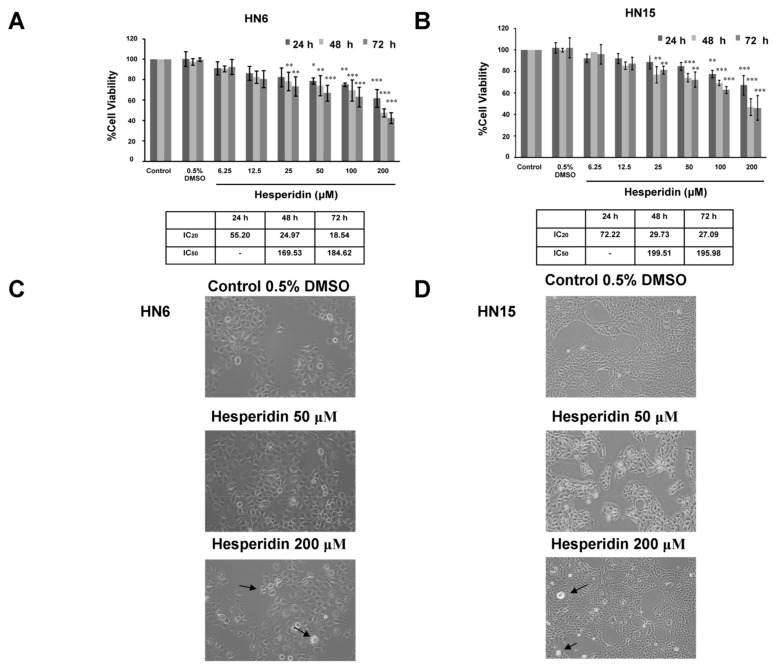
Viability and morphology of oral cancer cells upon treatment with hesperidin. The percentage of cell viability in HN6 (**A**) and HN15 (**B**) treated with hesperidin at indicated doses (0.25–200 μM) for 24, 48, and 72 h. Data are expressed as mean percentage values of viable cells relative to those of the control untreated cells that were set to 100. Error bars = standard errors; *n* = 3; * *p* < 0.05; ** *p* < 0.01; *** *p* < 0.001. The inhibitory concentrations of treatment with hesperidin for 24, 48, and 72 h at 20% (IC20) and 50% (IC50) for HN6 and HN15 are summarized in subsequent tables. Morphological changes of HN6 (**C**) and HN15 (**D**), after treatment with hesperidin at 50 or 200 μM for 24 h, were observed under an inverted phase-contrast microscope. Magnification power was set to 200×. Black arrows indicate membrane blebbing.

To investigate the anticancer effects of hesperidin on HN6 and HN15, the cell viability upon treatment with hesperidin at various doses from 0 to 200 µM for 24, 48, or 72 h was first studied using the MTT assay. Treatment with hesperidin decreased cell viability in both dose- and time-dependent manners with significant reductions in the mean percentage values of cell viability detected at 50 µM for a 24 h incubation period in HN6 or at 25 µM for 48 and 72 h incubation periods in HN6 and HN15 (*p* < 0.05; Figure 4A and Figure 4B, respectively). Inhibitory concentrations of hesperidin at 50% (IC_50_) for HN6 at 48 and 72 h were 169.53 and 184.62 µM, respectively, which were determined to be lower than those for HN15 (199.51 and 195.98 µM at 48 and 72 h, respectively) indicating that HN6 is more sensitive to hesperidin than is HN15. Likewise, IC_20_ at 24 h for HN6 was lower than that for HN15 (55.20 versus 72.22 µM, respectively). Concentrations of hesperidin that were lower than IC_20_, i.e., ≤ 50 µM, were then selected for subsequent experiments. In addition, the morphology of HN6 and HN15 was monitored after exposure to hesperidin at 50 or 200 µM for 24 h. Some HN6 and HN15 cell lines were rounded up and detached from the culture vessel. These were readily seen upon treatment with hesperidin at 200 µM (black arrows in Figure 4C and Figure 4D, respectively).

### 2.5. Inhibition of Colony-Forming Capacity and Migration of Oral Cancer Cells by Hesperidin

**Figure 5:**
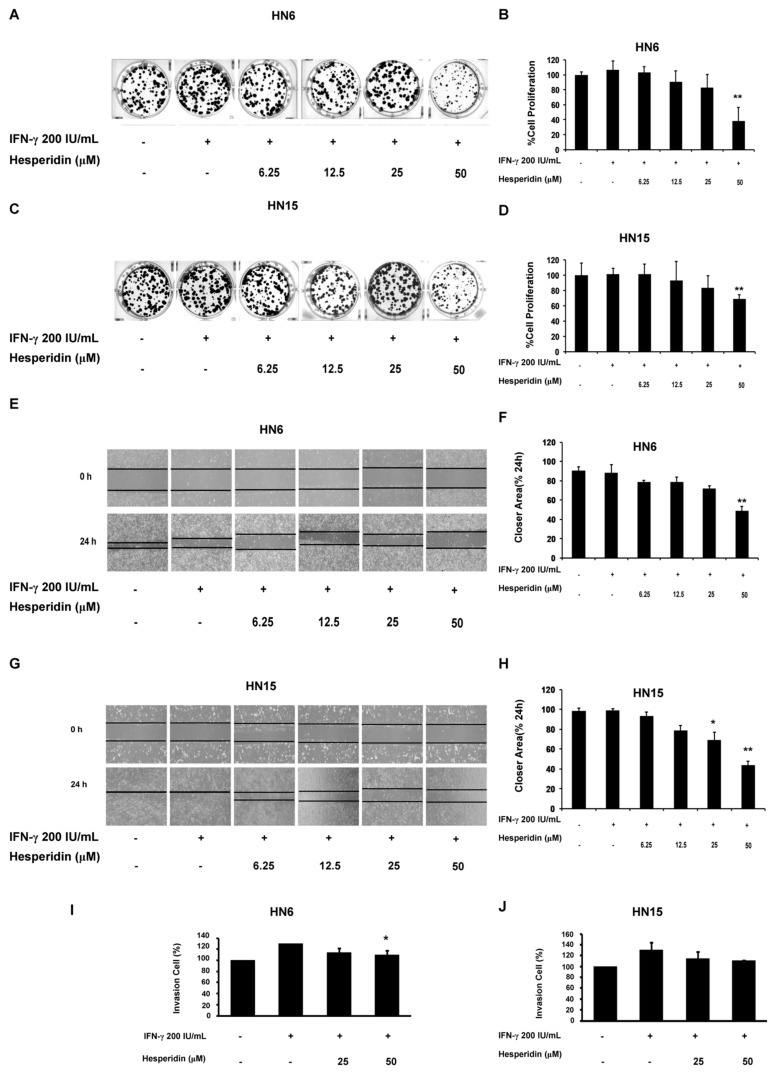
Anticancer effects of hesperidin on colony formation, migration, and invasion of oral cancer cells. Representative images of colony formation in HN6 (**A**) and HN15 (**C**) upon treatment with hesperidin at indicated doses (6.25–50 μM) in the presence or absence of IFN-γ at 200 IU/mL for 24 h. Bar graphs show significantly reduced percentages of cell proliferation in HN6 (**B**) and HN15 (**D**) upon treatment with hesperidin at 50 μM. Representative images of the wound healing assay revealed that treatment with hesperidin for 24 h inhibited the migration of HN6 (**E**) and HN15 (**G**). Bar graphs show significantly decreased percentage values of the closing area in HN6 (**F**) and HN15 (**H**) upon treatment with hesperidin at 50 µM. The invasion assay in HN6 (**I**) and HN15 (**J**) revealed that treatment with hesperidin at 50 µM significantly inhibited an increase in the percentage of invasion by IFN-γ treatment in HN6. Data in (**B**,**D**,**F**,**H**,**I**–**J**) are presented as mean ± SD values (error bars) obtained from three separate experiments. * *p* < 0.05; ** *p* < 0.01.

Next, the antiproliferative effect of hesperidin was determined using colony formation assay. The number of colonies was reduced by treatment with hesperidin in a dose-dependent manner with significant reductions in the mean percentage values of the colony numbers found at treatment with 50 µM of hesperidin in the HN6 and HN15 cell lines (*p* < 0.01; Figure 5A, Figure 5C, Figure 5B and Figure 5D, respectively). This implied that treatment with hesperidin could suppress the ability of both oral cancer cell lines to proliferate. Moreover, a wound healing assay revealed that cell migration was decreased by treatment with hesperidin in a dose-dependent manner with significant decreases in the mean percentage values of the closing area identified at treatment with 50 μM of hesperidin in HN6 (*p* < 0.01) and at treatment with 25 µM (*p* < 0.05) or 50 µM (*p* < 0.01) of hesperidin in HN15 (Figure 5E, Figure 5G, Figure 5F and Figure 5H, respectively). Furthermore, cell invasion assay revealed that treatment with hesperidin inhibited significant increases in the mean percentage values of cell invasion upon IFN-γ treatment in both HN6 and HN15 in a dose-dependent manner (Figure 5I and Figure 5J, respectively). A significant degree of inhibition was also observed upon treatment with hesperidin at 50 µM only in HN6 (*p* < 0.05; Figure 5I).

### 2.6. Inhibitory Effect of Hesperidin on IFN-γ-Induced PD-L1 Protein Expression in Oral Cancer Cells

**Figure 6:**
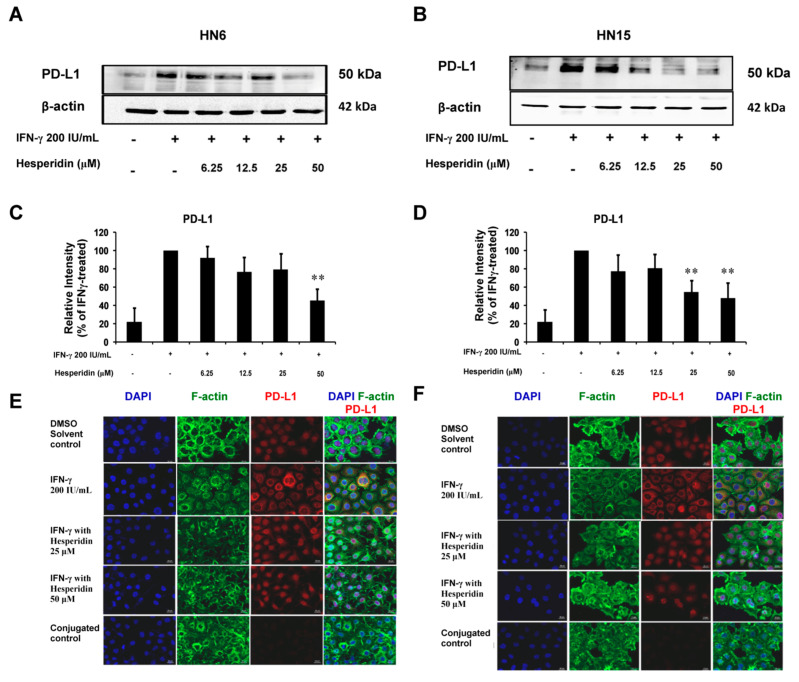
Downregulation of IFN-γ-induced PD-L1 expression by hesperidin in oral cancer cell lines. HN6 (**A**) and HN15 (**B**) were treated with hesperidin at indicated doses in the presence of IFN-γ at 200 IU/mL for 24 h. Bar graphs show the significant degrees of inhibition of induced PD-L1 by IFN-γ upon treatment with hesperidin at 50 µM in HN6 (**C**) and at 25 or 50 µM in HN15 (**D**). Data in (**C**,**D**) are presented as mean ± SD values (error bars) from three separate experiments. ** *p* < 0.01. Representative images from three experiments in HN6 (**E**) and HN15 (**F**) are shown. Cells were treated according to the method described above. Note the cytoplasmic staining (red) for PD-L1 protein, as indicated by the staining of F-actin (green) in the HN6 and HN15 cell lines, was induced by IFN-γ treatment at 200 IU/mL and then disappeared after treatment with hesperidin at 25 or 50 µM. Red nuclear staining in the control DMSO (a vehicle control for hesperidin), as indicated by DAPI staining (blue), is an artifact as has already been established in Figure 1B. HN6 or HN15, treated with IFN-γ at 200 IU/mL without incubation with the primary antibody against PD-L1, showed no red staining as a conjugated control.

To determine the effect of hesperidin on up-regulated PD-L1 expression by treatment with IFN-γ, HN6 and HN15 were treated with IFN-γ at 200 IU/mL in the presence or absence of hesperidin at various doses (6.25–50 µM) for 24 h. Through the process of immunoblotting, treatment with hesperidin decreased the level of up-regulated PD-L1 expression by IFN-γ in a dose-dependent manner in both HN6 and HN15 (Figure 6A and Figure 6B, respectively) with significant reductions in the mean percentage values of PD-L1 expression that were found after treatment with 50 µM of hesperidin in HN6 (*p* < 0.01; Figure 6C) and with 25 or 50 µM of hesperidin in HN15 (*p* < 0.01; Figure 6D). In addition, treatment with hesperidin at 25 or 50 µM abrogated the cytoplasmic fluorescence signal (red) of PD-L1 protein induced by treatment with IFN-γ at 200 IU/mL in HN6 and HN15 (Figure 6E and Figure 6F, respectively). These results indicate that treatment with hesperidin at doses lower than IC_20_ could effectively down-regulate PD-L1 expression that was induced by IFN-γ in both oral cancer cell lines. This suggests that the anticancer activities of hesperidin observed in Figure 4 and Figure 5 may be mediated by a decreased level of PD-L1 expression.

### 2.7. Inhibition of IFN-γ-Induced Phosphorylated STAT1 and STAT3 Levels by Hesperidin

**Figure 7:**
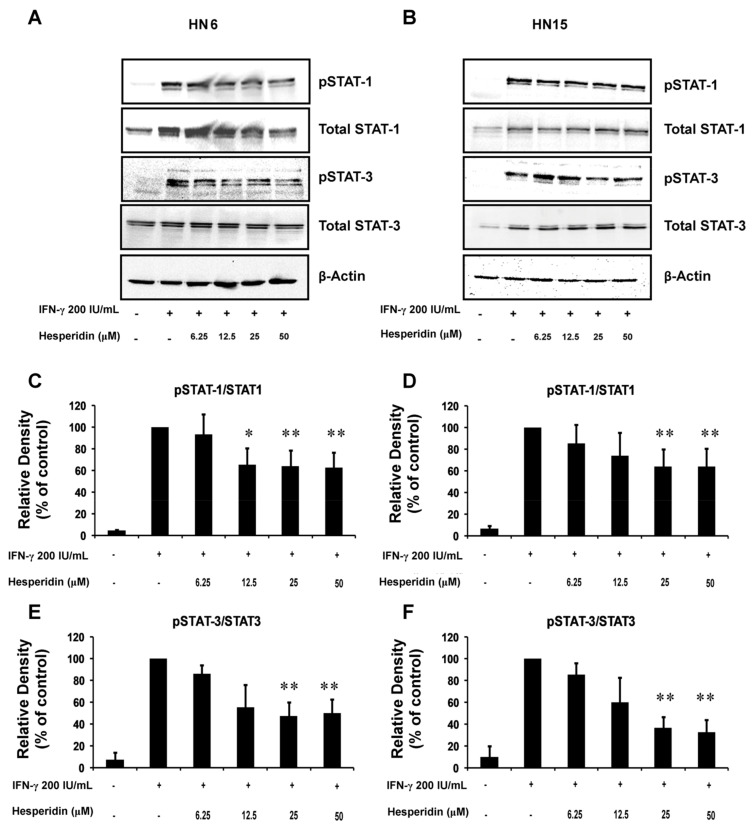
Inhibition of IFN-γ-induced phosphorylation of STAT1 (p-STAT1) and STAT3 (p-STAT3) by hesperidin in oral cancer cells. Representative immunoblots of p-STAT1, p-STAT3, total STAT1, and total STAT3 in the whole cell lysates of HN6 (**A**) and HN15 (**B**) are shown. Cells were pretreated with hesperidin at indicated doses for 4 h, followed by treatment with IFN-γ at 200 IU/mL for 30 min. Bar graphs show significant reductions in the percentage values of the relative intensities of p-STAT1/STAT1 or those of p-STAT3/STAT3 in HN6 (**C**) and (**E**), respectively and in HN15 (**D**) and (**F**), respectively upon treatment with hesperidin at 25 or 50 μM, as compared to those found in the IFN-γ treated samples set to 100. Data in (**C**–**F**) are presented as mean ± SD values (error bars) from three separate experiments. * *p* < 0.05; ** *p* < 0.01.

To gain further insights into the inhibitory mechanisms of hesperidin on PD-L1 expression via the IFN-γ signaling pathway, HN6 and HN15 were pretreated with hesperidin at indicated doses (0–50 µM) for 4 h. This was followed by treatment with IFN-γ at 200 IU/mL for 30 min. The levels of p-STAT1 and p-STAT3 were detected by Western blot hybridization. Pretreatment with hesperidin reduced the levels of p-STAT1 and p-STAT3 that were induced by IFN-γ at 200 IU/mL in a dose-dependent manner in HN6 and HN15 (Figure 7A and Figure 7B, respectively). By densitometry, the increased mean ratio of p-STAT1/total STAT1, and that of p-STAT3/total STAT3 by IFN-γ treatment, were significantly inhibited by pretreatment with hesperidin at 25 or 50 µM in HN6 (*p* < 0.01; Figure 7C and Figure 7E, respectively) and in HN15 (*p* < 0.01; Figure 7D and Figure 7F, respectively). These findings suggest that treatment with hesperidin could potentially inhibit up-regulated PD-L1 protein expression by IFN-γ via the STAT1 and the STAT3 signaling molecules.

## 3. Discussion

Unlike PD-L1 expression, detected as an immunoreactive band at 50 kDa or signal localized within the cytoplasm of the breast cancer cell line, MDA-MB231, the present in vitro study has demonstrated very low to no PD-L1 expression in the two oral cancer cell lines, namely HN6 and HN15. The discrepancy in PD-L1 expression between the oral and the breast cancer cell lines implies a unique feature for the molecular pathogenesis of oral cancer. However, treatment with exogenously added IFN-γ, a pro-inflammatory cytokine found within the tumor microenvironment, could significantly induce the expression of PD-L1 in a dose-dependent fashion in both oral cancer cell lines. This could possibly have occurred via the transient phosphorylation of STAT1 and STAT3, two major signaling molecules mediating the signal transduction of IFN-γ. Furthermore, although the cytoplasmic localization of PD-L1 protein was not detected in the untreated HN6 or HN15 cell lines, as assayed by immunofluorescence, the PD-L1 protein was found to be localized in the cytoplasm of IFN-γ-treated HN6 and HN15 cell lines. It is likely that the immunoreactive signal found in the nuclei of HN6 and HN15 is an artifact that results from inappropriate cell fixation and permeabilization during an immunocytochemical study as has been previously suggested [25].

Regardless of whether treatment with IFN-γ was administered, the PD-L1 protein had been already found to be expressed in several types of untreated cancer cell lines [26,27]. The absence or the presence of a faint immunoreactive band at the predicted size in untreated HN6 or HN15 cells, respectively, was initially rather surprising to us. Nevertheless, the unexpected immunoreactive bands at around 70–95 kDa were instead detected by immunoblotting in HN6 and HN15. It is possible that these bands are the ubiquitinated PD-L1 proteins, as has been previously demonstrated, at a molecular weight greater than 64 kDa [24]. In addition, it has been lately demonstrated that the PD-L1 protein can indeed undergo ubiquitination in oral squamous cell carcinoma [28]. The issue of posttranslational modification of the PD-L1 protein by ubiquitination in HN6 and HN15 has not yet been addressed in this study. Nevertheless, another previous study has suggested that inflammation could increase PD-L1 expression via the COP9 signalosome complex subunit 5 (CSN5), an essential regulator of the ubiquitin conjugation pathway, by decreasing the ubiquitination of the PD-L1 protein. This could lead to stabilization of the PD-L1 protein [29]. Therefore, it is probable that treatment with IFN-γ may decrease PD-L1 ubiquitination in HN6 and HN15 resulting in more intact PD-L1 protein being detectable at its predicted size. This subject remains to be further explored. Several previous studies have demonstrated that treatment with IFN-γ induces the expression of PD-L1 in various types of cancer cells such as breast cancer, lung cancer, and oral cancer cells [10,23,30]. Moreover, it has been well-established that a higher degree of PD-L1 protein expression is associated with immune escape and metastasis in different types of cancer cells [31,32]. The findings from this in vitro study, which demonstrated significant increases in PD-L1 expression as well as in cell invasion upon treatment with IFN-γ in the oral cancer cell lines, HN6 and HN15 (Figure 5I,J), are thus in line with those of a previous study [33] that emphasized the significant role of PD-L1 in cancer aggressiveness.

**Figure 8:**
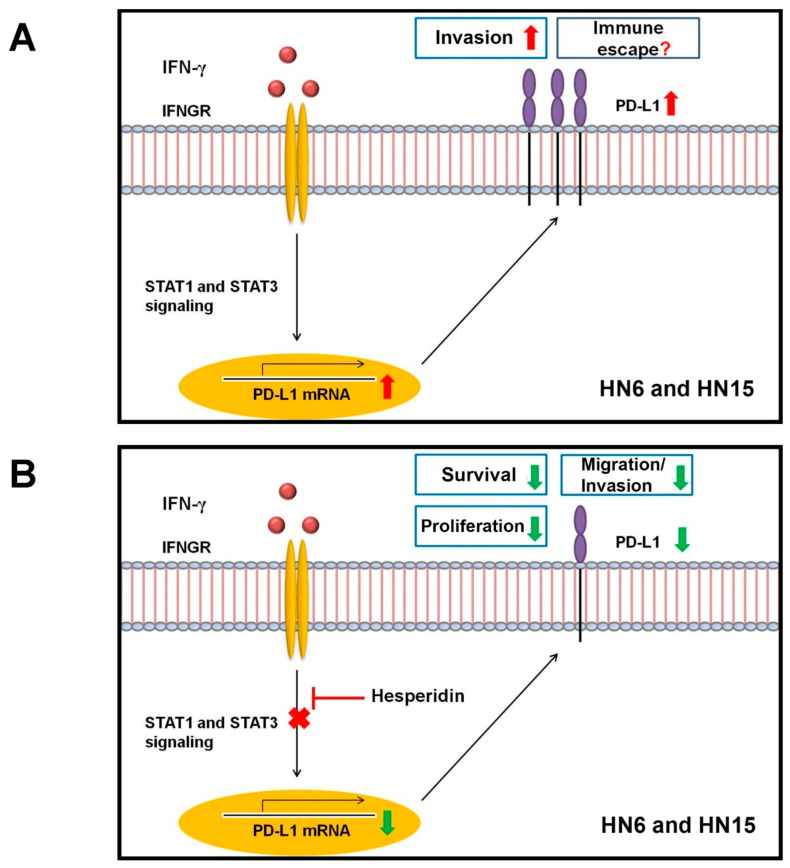
Schematic diagrams illustrating (**A**) an increase in the invasion of certain oral cancer cell lines, including HN6 and HN15, upon stimulation with interferon-gamma (IFN-γ). This likely resulted from the activation of the signal transducer and activator of the transcription (STAT) 1 and 3 signaling molecules and from an increase in the amount of programmed death-ligand 1 (PD-L1) expression. However, a possible escape from immune surveillance by increased PD-L1 expression in HN6 and HN15 has not yet been determined, while (**B**) a proposed mechanism for the anticancer effects of hesperidin on decreased oral cancer cell survival, proliferation, migration, and invasion could have occurred by inhibiting phosphorylation of STAT1/STAT3 and then suppressing PD-L1 expression. IFNGR = interferon gamma receptor.

In this study, treatment with hesperidin at doses lower than IC_20_, i.e., ≤50 µM, could significantly diminish oral cancer cell survival, proliferation, migration, and invasion. This probably would have occurred as a result of reduced STAT1/STAT3 activation followed by decreased PD-L1 expression upon hesperidin treatment (Figure 8). Nonetheless, the direct roles of PD-L1 in the four aspects of oral cancer aggressiveness, as has been aforementioned, as well as in the promotion of an evasive mechanism that oral cancer cells, especially HN6 and HN15, exploit in order to escape immune surveillance have not yet been determined (Figure 8). Consequently, these roles should be the subject of further investigations. Otherwise, an additional investigation into enhanced tumor immunity, which leads to the inhibition of immune evasion and cancer aggressiveness, by suppressing the up-regulated expression of PD-L1 via the reduced activation of STAT1/STAT3 signaling, is worth pursuing in oral cancer cell lines.

The development of natural compounds as an adjunctive treatment for cancer is of great interest. Any natural compound that possesses an anti-inflammatory property and can block or regulate the inducible expression of PD-L1 protein is thus suitable as a therapeutic candidate for an immune checkpoint inhibitor [34]. In this regard, it has recently been reported that hesperidin acts as an essential regulator of PD-L1 expression in the breast cancer cell line [23]. Consequently, it is of considerable interest to determine the anticancer effects of hesperidin and evaluate the regulation of PD-L1 expression in human oral cancer cell lines in vitro. Firstly, the findings from the cytotoxic screening showed that treatment with hesperidin inhibited the viability of the two oral cancer lines in dose- and time-dependent fashions. In addition, from the previous study, they found this concentration (0–200 µM) of hesperidin does not affect normal cells such as the human dermal fibroblast (NHDF) cell line [35] and the normal liver cell line [36]. However, it is reported that hesperidin suppresses cell proliferation in several cancer cells types. Secondly, our findings revealed that treatment with hesperidin was able to partially decrease the cell proliferation, migration, and invasion of these oral cancer cell lines. Finally, treatment with hesperidin partially diminished IFN-γ-induced PD-L1 expression and the phosphorylation of STAT1 and STAT3. Since STAT1/STAT3 signaling is regarded as one of the critical pathways for cell proliferation, migration, and invasion [36,37], it is likely that the anticancer effects of hesperidin are mediated by STAT1/STAT3 signaling and activated by treatment with IFN-γ in the oral cancer cell lines. Nevertheless, due to the partial reduction of STAT1/STAT3 phosphorylation upon treatment with hesperidin, it is, therefore, necessary to elucidate the involvement of other signaling pathways.

Hesperidin has been shown to have anti-cancer effects in different malignancies, emphasizing its molecular mechanism of action. Hesperidin acts as an anti-cancer agent by promoting apoptosis in malignant cells such as liver cancer and bladder cancer cells via NF-κB, MAPK, and PI3K/AKT pathways. Moreover, hesperidin inhibits the expression of MMP and epithelial-mesenchymal transition (EMT)-related proteins, suppressing cell migration and invasion, as well as being an anti-inflammatory [34]. This study discovered that hesperidin prevents IFN-γ-induced PD-L1 protein expression by inactivating STAT1/STAT3 signaling in OSCC cancer cells, contributing to tumors’ immune evasion. In addition to the partial blockade of STAT1/STAT3 activation by treatment with hesperidin, their off-target effect is still questionable. To address the potential off-target problem of STAT1 and STAT3 in IFN-γ-induced PD-L1 expression in the oral cancer cell lines, knockdown of the STAT1/STAT3 expression with specific siRNAs would be required. In addition, the effect of hesperidin on signaling pathways other than STAT-1 and STAT-3 in OSCC cell types has not been explored. Other reports have indicated that often signaling involves PD-L1 up-regulation, including in NF-κB, MAPK, and PI3K/AKT pathways. In future research, the other signaling mechanisms for the effect of hesperidin should be explored. Moreover, the efficacy and the safety of standard chemotherapy in combination with hesperidin treatment in a responsive syngeneic tumor model are essential issues of concern. Before hesperidin can be applied in clinical trials, which may offer a novel treatment option for patients with oral cancer, additional in vivo studies are required to confirm the inhibitory effect of hesperidin on the inducible PD-L1 expression by IFN-γ. The effect of hesperidin on T cell activity should also be investigated both in vitro and in vivo. Lastly, the insight gained from the present study could enable researchers to propose the potential clinical application of hesperidin as an immune checkpoint inhibitor in the future.

## 4. Materials and Methods

### 4.1. Chemical Reagents and Antibodies

Serum-free keratinocyte growth medium (KGM) was obtained from Lonza (Walkersville, MD, USA). Fetal bovine serum (FBS), phosphate-buffered saline (PBS), and trypsin-EDTA solution were purchased from Gibco (Grand Island, NY, USA). Hesperidin, 3-(4,5-dimethylthiazol-2-yl)-2,5-diphenyltetrazolium bromide (MTT), and dimethyl sulfoxide (DMSO) were purchased from Sigma Chemical, Inc. (St Louis, MO, USA). Furthermore, 4′,6-diamidino-2-phenylindole (DAPI) was obtained from Biotium, Inc. (Hayward, CA, USA). Mammalian Protein Extraction buffer was purchased from GE Health Care Life Sciences (GmbH, Freiburg, Germany). Primary antibodies against human PD-L1, phosphorylated-STAT1 (p-STAT1), total STAT1, p-STAT3, total STAT3 or β-actin, and anti-rabbit horseradish peroxidase (HRP)-conjugated immunoglobulin G (IgG) were purchased from Cell Signaling Technology, Inc. (Beverly, MA, USA). A Super Signal West Femto Maximum Sensitivity substrate kit Pico and Alexa Fluor™488-conjugated Phalloidin were obtained from Thermo Fisher Scientific, Inc. (Waltham, MA, USA). Protease and phosphatase inhibitors were obtained from Roche Diagnostics (Mannheim, Germany). Recombinant human IFN-γ and anti-rabbit NorthernLights™557-conjugated IgG were purchased from R&D Systems, Inc. (Minneapolis, MN, USA). Hesperidin was dissolved in DMSO and stored at −20 °C until used. The final concentration of DMSO in all experimental samples was <0.5% (*v*/*v*).

### 4.2. Oral Cancer Cell Lines and Cell Cultures

The two human OSCC cell lines used in this study were HN6 and HN15. HN6 was originally isolated from the tongue of a male patient with OSCC with T_2_N_0_M_0_ staging who had received a histopathological diagnosis of acquiring moderately differentiated OSCC [35]. HN15 was isolated from the metastatic lymph node with the primary OSCC site established on the floor of the mouth [36]. These cells were cultured in serum-free KGM supplemented with 1% penicillin/streptomycin at 37 °C in a humidified atmosphere containing 5% CO_2_. When cells reached 80% confluence, they were trypsinized and plated in appropriate culture vessels for either expansion of their cell numbers or further experimentation.

### 4.3. Cytotoxic Assay

HN6 and HN15 (5000 cells/well) cells were seeded in a 96-well culture plate overnight and then exposed to various doses (0, 6.25, 12.5, 25, 50, 100, and 200 µM) of hesperidin for 24, 48, or 72 h. Cell viability was determined by MTT assay as has been previously described [23]. The optical density (OD) of dissolved formazan dye was measured using a spectrophotometric plate reader (Thermo Fisher Scientific, Inc.) at 540 nm with a reference wavelength of 630 nm. The percent of cell viability (% of cell viability) was calculated as the sample/OD of control × 100.

### 4.4. Colony Formation Assay

The antiproliferative effect of hesperidin on HN6 and HN15 was examined using a colony formation assay. Briefly, HN6 and HN15 at 8 × 10^2^ cells/well were seeded in a 6-well culture plate overnight. The cells were treated with IFN-γ at 200 IU/mL in the presence or absence of hesperidin at 6.25, 12.5, 25, or 50 µM for 24 h. Next, KGM was removed and replaced with fresh KGM every other day to allow for colony formation over the course of 2 weeks. The colonies were washed with cold PBS, fixed with 95% ethanol for 15 min, and stained with 0.5% crystal violet for 1 h at room temperature. Images of the stained colonies were captured with a digital camera attached to a microscope and 10% (*v*/*v*) of acetic acid was then added to each well. This was followed by measurement of the absorbance value of the dissolved dye at 595 nm using the spectrophotometric plate reader.

### 4.5. Immunofluorescence

HN6, HN15, and MDA-MB231 as a control cell line, were seeded in glass chamber slides (Lab-Tek^®^ Chamber Slide™, Nunc, Rochester, NY, USA) at 4 × 10^4^ cells/200 µL overnight. HN6 and HN15 were then treated with IFN-γ at 200 IU/mL (Figure 2) in the presence or absence of treatment with hesperidin at 25 or 50 µM (Figure 6) for 24 h, or the three cell lines were left untreated (Figure 1). Immunofluorescence staining of PD-L1 was performed the following day. In brief, cells were washed with 1X PBS, fixed with 4% paraformaldehyde for 40 min, and solubilized with 0.1% (*v*/*v*) Triton X-100 in 3% (*w*/*v*) bovine serum albumin–PBS for 2 min. They were then reacted with the PD-L1 antibody (1:200) in PBS without any detergent at 4 °C overnight. After being washed, they were incubated with anti-rabbit NorthernLights™557-conjugated IgG (1:500), Alexa Fluor™488-conjugated Phalloidin (1:500), and DAPI (1:1.000) in PBS at room temperature for 1 h. The slides were then mounted with DAKO^®^ Fluorescent Mounting Medium (DAKO Corporation, Carpinteria, CA, USA). The fluorescence signals were observed and captured under a fluorescence microscope (Axio with ApoTome.2, Carl Zeiss Microscopy GmbH, Göttingen, Germany). As can be seen in Figure 6, HN6 and HN15 treated with 0.5% (*v*/*v*) DMSO were used as a vehicle control for hesperidin. Subsequently, the cells treated with IFN-γ at 200 IU/mL for 24 h in the absence of immunoreaction with the PD-L1 antibody were used as a conjugated control.

### 4.6. Immunoblotting

To determine PD-L1 expression and p-STAT1 and p-STAT3 levels, HN6 and HN15 seeded at 2 × 10^5^ cells/well in a 6-well plate were treated with indicated doses (0–400 IU/mL) of I IFN-γ for 24 h (Figure 2) or with IFN-γ at 200 IU/mL for various periods of time (10–60 min and 24 h; Figure 3). To elucidate the involvement of the PD-L1 expression and the p-STAT1 and p-STAT3 levels, these cell lines were treated with IFN-γ at 200 IU/mL in the presence or absence of treatment with hesperidin at 6.25, 12.5, 25, or 50 μM for 24 h (Figure 6), or were pretreated with hesperidin at 6.25, 12.5, 25, or 50 μM for 4 h, followed by treatment with IFN-γ at 200 IU/mL for 30 min (Figure 7). The cells were then lysed in Mammalian Protein Extraction buffer containing both protease and phosphatase inhibitors. Total protein content was determined using the Bradford assay. A 30 µg quantity of total protein obtained from each sample was resolved on 12% SDS-PAGE, according to the method previously described [37], and then transferred to nitrocellulose membranes (GE Healthcare Europe GmbH, Freiburg, Germany). The membranes were blocked with 5% (*w*/*v*) skim milk in Tris-buffered saline with Tween-20 (TBST) for 1 h at room temperature. The membranes were then washed three times with TBST and probed overnight with specific primary antibodies against p-STAT1, total STAT1, p-STAT3, total STAT3, PD-L1, or β-actin at a dilution factor of 1:1.000 at 4 °C. After being washed three times, the membranes were exposed to an appropriate secondary antibody for 1 h at room temperature. After three additional washings with TBST, the membranes were allowed to react with an enhanced chemiluminescence substrate (Super Signal West Femto) in order to develop protein bands that were captured using the ChemiDoc XRS system (Bio-Rad Laboratories, Hercules, CA, USA). The band intensity was then analyzed using ImageJ software. After one target protein was detected, the primary and secondary antibody complex was removed using the stripping buffer (Thermo Fisher Scientific, Inc.) for 15 min. Subsequently, the membrane was re-probed with the antibody against the other protein of interest and the intensities of the protein bands were again determined. The intensity of β-actin band in each sample was used as an internal control for PD-L1 expression, while that of total STAT1 or of total STAT3 was used as an internal control for p-STAT1 or p-STAT3, respectively.

### 4.7. Wound Healing Assay

HN6 and HN15 were cultured in a 6-well plate. At a level of 80% confluence, cells were scratched using a 200 μL pipette tip and washed with PBS. Thereafter, the cells were cultured with serum-free KGM and treated with IFN-γ at 200 IU/mL in the presence or absence of hesperidin at amounts of 6.25, 12.5, 25, or 50 μM for 24 h. Images of the closing area were captured with a camera attached to a microscope (Carl Zeiss Microscopy GmbH) both at the beginning (0 h) and at the 24 h timepoint. The percentage of closing area within the space between the wound edges was calculated using ImageJ software.

### 4.8. Invasion Assay

HN6 and HN15 at 2 × 10^5^ cells/mL were seeded in a 24-well culture plate for 48 h. At 80% confluence, cells were treated with IFN-γ at 200 IU/mL in the presence or absence of hesperidin at 25 or 50 µM for 24 h. The Cell Detachment Solution prepared from the fluorometric QCM™ 24-Well Cell Invasion Assay kit (Merck, Darmstadt, Germany) was then added into each well to lift each of the cells, whose number was first counted and adjusted to 5 × 10^5^ cells/mL. The invasion assay was performed using the fluorometric QCM™ 24-Well Cell Invasion Assay. In brief, cell inserts were rehydrated with 300 μL of prewarmed KGM for 30 min at room temperature. After removal of the medium, the insert was filled with a 250 μL volume of KGM, which contained HN6 or HN15, and the lower chamber was filled with only 500 μL of KGM. The invasion assay was performed in a CO_2_ incubator for 14 h. The cells and medium collected from the top side of the invasion chamber insert were removed through careful pipetting and the insert was placed into a clean well containing 225 μL of the prewarmed Cell Detachment Solution. The resulting cell mixture was incubated for 30 min at 37 °C. The invasive cells were dislodged from the underside of the insert by gently tiling the invasion chamber plate back and forth several times. After removal of the insert, 75 μL of the Lysis Buffer/Dye Solution obtained from the kit was added and the cell mixture was then incubated for 15 min at room temperature. The cell mixture at a volume of 200 μL was transferred to a 96-well black plate with a clear bottom. The degree of fluorescence intensity was measured using a multi-mode microplate reader (Spark, Tecan Austria GmbH, Grödig, Austria) at 480 nm excitation and 520 nm emission wavelengths. The fluorescence signal of each condition was compared to that of the untreated cells of the control set to 100% of invasion.

### 4.9. Statistical Analysis

Each experiment was repeated independently three times. Data were expressed as mean ± SD values of the three experiments. Statistical analysis was performed using SPSS 12.0 software and one-way ANOVA followed by Tukey’s HSD post hoc test. Additionally, *p* values of < 0.05 were considered statistically significant.

## Data Availability

Not available.

## Sample Availability

Samples of the compounds are not available from the authors.
